# From face detection to emotion recognition on the framework of Raspberry pi and galvanic skin response sensor for visual and physiological biosignals

**DOI:** 10.1186/s43067-023-00085-2

**Published:** 2023-04-18

**Authors:** Varsha Kiran Patil, Vijaya R. Pawar, Shreiya Randive, Rutika Rajesh Bankar, Dhanashree Yende, Aditya Kiran Patil

**Affiliations:** 1 Department of Electronics and Telecommunication Engineering, AISSMS Institute of Information Technology, Pune, India; 2 Department of Electronics and Telecommunication Engineering, Bharati Vidyapeeth College of Engineering for Women, Pune, India; 3 Department of Electronics and Communication Engineering, BITS Pilani Goa Campus, Goa, India

**Keywords:** Galvanic skin response, Raspberry pi, Face detection, Machine learning, Emotion recognition, Facial emotion recognition, Affective computing, Computer vision, Psychology, Haar cascade, Human–machine interface (HMI), Data fusion, Physiological signal, Habitation test, Video induction, Stimuli, Correlation matrix, Physiological signal-based emotion recognition, Sensovisual, Emosense

## Abstract

The facial and physiological sensor-based emotion recognition methods are two popular methods of emotion recognition. The proposed research is the first of its kind in real-time emotion recognition that combines skin conductance signals with the visual-based facial emotion recognition (FER) method on a Raspberry Pi. This research includes stepwise documentation of method for automatic real-time face detection and FER on portable hardware. Further, the proposed work comprises experimentation related to video induction and habituation methods with FER and the galvanic skin response (GSR) method. The GSR data are recorded as skin conductance and represent the subject's behavioral changes in the form of emotional arousal and face emotion recognition on the portable device. The article provides a stepwise implementation of the following methods: (a) the skin conductance representation from the GSR sensor for arousal; (b) gathering visual inputs for identifying the human face; (c) FER from the camera module; and (d) experimentation on the proposed framework. The key feature of this article is the comprehensive documentation of stepwise implementation and experimentation, including video induction and habituation experimentation. An illuminating aspect of the proposed method is the survey of GSR trademarks and the conduct of psychological experiments. This study is useful for emotional computing systems and potential applications like lie detectors and human–machine interfaces, devices for gathering user experience input, identifying intruders, and providing portable and scalable devices for experimentation. We termed our approaches "sensovisual" (sensors + visual) and "Emosense" (emotion sensing).

## Introduction

Human activities and productivity, interpersonal relations, and reactions have been influenced by emotions. Recent developments in the emotion recognition field are because of advanced software algorithms, the availability of hardware with high-speed communication capabilities [1], and the optimized design of Human Machine Interaction (HMI) systems [2].

Figuring out how to implement real-time automatic emotion recognition is a complex and crucial problem because emotions are subjective to an individual’s ability to react to different stimuli. Visual observations of the faces in various situations indicate different facial emotions. Positive emotions can be recognized from smiles and happiness on faces [4]. Negative emotions and undesirable effects by humans can be recognized with Sadness, disgust, neutral faces. The main drawback of Facial emotion recognition is that some people may hide faces or some may be expert in hiding underlying emotions.

Biosignals from the skin can be used to determine emotions because it is the primary interface between the environment and the organism. Several studies have been conducted on sweat biomarkers and their role in wearable devices for emotion recognition [5]. Triggers cause the process of releasing moisture through the skin. Sweat gland moisture causes differences in skin conductance. The difference in skin conductance is sensed by Galvanic Skin Response (GSR) in the form of change in conductance. Emotional fluctuations can be detected when sweat ducts fill, causing changes in skin conductance [6]. Such reactions can provide useful information about emotional stimulation. [6] presenting gaming-related responses and skin basics that cause bio parameter changes.

Physiological-based emotion recognition is the best alternative to facial emotion recognitions (FER). Changes in the biosignals in response to the changes in emotions are indicators of the changes in the automatic nervous system. As a result, these insuppressible biosignals from the subjects can be utilized as the true indications of the underlying emotions [4]. This trend is useful for use of the wearable technologies in the affective computing domain. Further, the proposed setup is useful for experimentation in an affective domain because it has advantage of portability and flexibility. While implementing the proposed research, the following research questions (RQ) were considered:

- *RQ1*: What are the prominent biosensors for emotion recognition and why we will choose skin biosignals?
- *RQ2*: Can we implement emotion recognition with the combination of the use of skin biosignals along with facial emotion recognition?
- *RQ3*: Can we propose a combinational approach of skin biosensor and visual method to be implemented on a portable device?

Table 1 provides comparison of biosignal methods. The research question RQ1 can be answered in the best way by comparisons as shown in Table 1. We have chosen GSR for its fast response and less setup time. Table 1 is useful for researchers choosing methods as per their applications. It provides guidelines for choosing one of the methods for extracting physiological signals for emotion recognition. Answer to RQ2 and RQ3 are provided in the “Method section” in a stepwise manner.

**Table 1:** Comparison of biosignal methods

S. no.	Method	Electrodes	Placement of the signal-capturing device	Processing	Advantage disadvantage
1	Electroencephalogram (EEG) sensors	Fixed method and variable method 12 electrodes is one of the popular methods	10–5,10–10, 10–20 methods at right ear, from front to back Frontopolar, frontal, central, parietal and occipita((o). Left are odd numbered and even are placed at the right part of the head Middle is called zero line	Wavelets. Machine learning and deep learning algorithms, signal processing methods, hybrid methods	Other fields of applications for proposed system are Human Computer Interface, medical dignosis and military field. Nonstationary and nonlinearity are drawbacks
2	Electrocardiogram (ECG) sensors	Twelve lead is popular. For laboratory conditions,3 leads are used,	For laboratory conditions, three lead ECG with the Right arm, left arm and left leg	NN50, SDNN, RMSSD are some of the features	improvisation in the previous research has hurdles of Age group bias, subjectivity in readings
3	Electromyography (EMG)	Surface Noninvasive EMG, and intramuscular	Facial EMG near the chin is popular. Low cost Myosensor is available	Methods like KNN, SVM are useful	Research is ongoing
4	Galvanic Skin Response sensors	Epidermal sensors	Mostly electrodes at fingers, palm, and feet. Useful in laboratory environment	AI and statistical methods can be used in combination of photoplethysmogram (PPG), EEG, and EEG	Advantage is fast response of emotions from skin. Disadvantage is the readings may deviate due to temperature and humidity

## About proposed work

The proposed galvanic framework in this article is proposed after the following observations.

- Facial emotions may be miscommunicated or may be manipulated by the subject/participant. Underlying emotions on faces may be fake and may be fabricated by the subjects.
- Changes in emotions cause changes in biosignals.
- The changes in the biosignal readings are difficult to mask or manipulate. Hence, the changes in the biosignals can represent underlying emotions and if both facial and biosignals are observed in one window (simultaneously), it will an advantageous, novel, and useful method.

### Objectives of Proposed work

The objectives of this article are as follows:

1. To document the systematic process of implementing raspberry pi-based face and GSR biosensor-based emotion detection.
2. To combine GSR-based arousal detection with a framework for facial expression analysis and then identify emotion and arousal using a combined method with videos as stimuli.
3. To experiment on the proposed framework for observing different psychological effects by experimentation done by habituation effect and video induction method.

With these objectives, we have proposed a novel combination of automatic emotion recognition, real-time computer vision, and sensor-based emotion recognition. Our proposed method is novel because this is first time, a combinational method of skin sensor and facial expression identification for psychological testing is done on the portable platform. The major contribution of the article is providing psychological platform experimentation and stepwise instructions for implementing the proposed framework dealing with real-time biosignal experimentation. We have named our proposed "sensovisual Emosense," where "sensovisual" stands for the combination of the camera and biosensor. 'Emosense' stands for “sensing emotions of subject”.

## Organization of the article

The introduction section poses thoughts that lead researchers to ask questions.  Introduction section also poses the novelty of proposed method  and describes the organization of the remaining sections of the article. The "Related Work" discusses the market and literature survey and system components. In the "Methods" section, the sequential process, algorithms, and flowcharts are described. In the section under "Results and Discussion," the outcomes of experiments and graphs are presented. The "Conclusion" section summarizes the article by discussing the sensovisual model's uses, strengths, future scope, and limits.

## Related work

Automatically recognizing the accuracy of human emotions has been a difficult task, but many researchers have recently started to work on it. In this section, we are presenting a market and literature survey for emotion recognition with visual and sensor methods. Although advances in computer vision algorithms and libraries facilitated in the human face and emotion recognition, the researchers also chose other methods for emotion detection. Attempts have been made in recent years to detect and validate emotions using various biosensors such as BPM ECG, GSR, EMG, and EEG. Table 1 shows comparison of various biosensors. We have chosen galvanic skin Response sensor. The GSR is good to obtain in controlled laboratory conditions. Unlike other methods, GSR is having less electrode arrangements and has fast response. Our proposed "Sensovisual Emosense" method combines visual and GSR sensor methods.

### Market survey

According to the market survey, GSR devices with wireless capabilities are available, but they do not support facial expressions at the same time. The devices in the market survey are expensive. A market survey reveals the need for a low-cost combined sensovisual method for emotion recognition. GSR represents the basic idea behind the device's changes in the form of changes in electrical body parameters.

Table 2 shows commercially available GSR devices. Each one has its advantages disadvantages and characteristics. The Empatica is a wristband-like Bluetooth hardware support. The AD Instruments Power lab GSR module and the Neulog GSR (Nul 217 GSR) provide Wi-Fi/wireless communication capabilities. R notebook support is provided by iMotions. Leading available trademarks include BioPAC GSR 100 C, Brain support, and MSP laboratory. The Groove GSR has been tested with Embedded C and microcontrollers.

**Table 2:** A market survey of available galvanic skin sensor trademark models

S. no.	GSR models name	Software	Support	Specifications/remarks
1	BioPAC GSR 100 C	Labpac, psychology	Other body sensors support are also available, superlab stimuli, event trackers, streaming, wireless monitoring	Output is 0 to 50 micromho, sensitivity 0.7 nano mho
2	AD Instruments Powerlab	LabChart	Wireless support	Powerlab has other body sensors that support a range of sensors
3	iMotions GSR module	Emotions R library from GitHub	R notebooks	GSR peak, signal quality. GSR epoch
4	MSP Lab	Shimmer 3 GSR and iMotions software	Biosemi active two GSR kit	Biosemi has ANT Neuro MyLab GSR pack containing an accelerometer and temperature sensor
5	Groove GSR (selected for experimentation)	Embedded C $$\text{Human resistance}=(1024+2*\text{Serial port value}*10000 ) / (512-\text{serial port reading})$$	Skin resistance is the output (unlike skin conductance in other cases)	Works with a kind of Arduino called Seeeduino and raspberry pi
6	Neulog GSR:Nul 217 GSR	Plug and play modules of GSR and all other sensors	Wireless support	0 to 10 microseconds Range, ADC resolution 16 bit, Resolution 10 ns
7	Empatica	Software available after the Online account to sync data to E4 manager	Bluetooth (BLE) support	Hardware that looks like Wristwatch
8	Brain support products	Electrical conductance is the output recorded by the bipolar amplifier	Constant dc voltage 0.5 voltage	1–30 microsecond range

Out of the commercial GSR devices mentioned in Table 2, we have chosen Groove galvanic sensor in our proposed method. The important reason for choosing is availability, inexpensiveness and flexible interface as compared to other commercially available methods. Groove GSR sensor provides mathematical relation for the Serial output parameter reading. As demonstrated in further sections, we can code the logic for emotion recognition with Grove GSR using Embedded C language. According to the market survey, GSR devices with wireless capabilities are available, but they do not support facial expressions at the same time. The devices in the market survey are expensive. A market survey reveals the need for a low-cost combined sensovisual method for emotion recognition. Groove GSR represents the basic idea behind the device's changes in the form of changes in electrical body parameters.

### Literature survey

This article uses GSR sensors for emotion recognition. Skin interacts with the sweat from the body in response to the emotion changes. Several studies have been conducted on sweat biomarkers and their role in wearable devices for emotion recognition [5]. Different kinds of stimuli cause the process of releasing moisture through the skin. Sweat gland moisture causes differences in skin conductance. This difference in skin conductance is known as GSR. Emotional fluctuations can be detected when sweat ducts fill, causing changes in skin conductance [6]. Such reactions can provide useful information about emotional stimulation. Article [6] presents gaming-related responses and skin basics that cause bio parameter changes. Hence, automatic emotion recognition on the portable platform is a very significant step in the affective computing domain.

Serrano et al. [7] proposed a novel method for recognizing human emotions based on heart rate (HR), body temperature (BT) and galvanic skin reaction (GSR). The method [7] is useful for collecting body parameters with a portable device and could be used to detect emotions. Paul et al. [8] mentioned the method of studying the human emotional condition. The emotional states were observed for a set amount of time in this paper. The methods and parameters used to detect emotional states in their work were GSR, BPM, body temperature, and EEG (Electroencephalogram) with ANOVA analysis, 'F', and 'MSE' tools.

The authors concluded that readings of body parameters could be used to distinguish between relaxed, operating, and reading mental states [8]. Villarejo et al. [9] developed an anxiety sensor that used the ZigBee protocol. Adults engaged in activities such as mathematical problem-solving and deep breathing were tested with the device using GSR. This GSR-based device had a success rate of 76.56%. The work included graphical responses to situations such as breathing, reading, and drinking coffee. Goshvarpour et al. [10] proposed a novel emotion classification method based on the collection and processing of ECG and GSR readings from 11 healthy students.

The wavelet packet dictionaries Coiflet and Daubechies were used. Matching pursuit coefficient extraction, dimension reduction, feature extraction, and neural network were additional steps for emotion classification related to subject-related mode [10]. Pantic et al. [11] published an article about computer recognition of facial expressions and human emotions. The article is a systematic review of previous articles on facial landmarks, which paved the way for real-time face and emotion detection. The authors discussed in detail the stages of development from P. Ekman's Facial Action Coding System to the characteristics, approaches, and applications of facial emotion recognition. Based on facial fluidical points, automatic face, and emotion detection, Henriques et al. [12] conducted experiments with 30 participants and 8 stimuli. They used Affectiva-QSensors5 technology to capture electro-dermal signals, and Symbolic Estimates to capture facial expression and body parameters. Hidden Markov-based models were used to classify emotions by selecting features and sequences.

Shu et al. [13] presented emotion detection based on physiological signals, which may be useful for use cases such as health care, safe driving, and social security. The paper provides a comprehensive overview of emotion recognition based on models, physiological data sets and cues, traits, classifiers, and a general framework for emotion recognition based on physiological signals.

According to Abadi et al. [14], the novel includes body parameter readings from fifty-eight participants as well as self-assessment reports. The authors used movie clips as inspiration. In previous observations, the authors experimented with associations between affective ratings and behavioral tendencies. The authors confirmed and concluded that emotions and relationships have a nonlinear relationship. Aldhahab et al. [15] proposed facial recognition technology with a feature extraction approach with various variations and precision. Recognizing a human face is a simple task for everyone, even if he has not seen the person in years. However, performing the same task with a computer is difficult because the computer has difficulty recognizing the human face when face images, conditions, complex backgrounds, poses, and occlusions change. Face Recognition System used 3D images of faces to recognize faces in images by using the nose as the peak point. This study's contribution is determining the maximum peak point in the images and determining the nose [15].

Ayatya et al. [16] achieved outstanding results for valence and arousal classification using time domain, based features, and wavelet features, as well as techniques such as the random forest. Empirical Mode Decomposition was also used. Ayatya et al. [16] used machine-learning algorithms such as the Support Vector Machine. The paper discusses various aspects of GSR-based emotion capture.

## Methods

The proposed research is about identifying human emotions using sensors and computer vision. The gradient boosting classifier algorithm is used for computer vision facial expression recognition on the raspberry pi microcontroller. The ATmega 32 microcontroller processes the galvanic sensor readings and sends them to the Raspberry Pi.

The steps below show how to design an emotion recognition system using the Raspberry Pi and ATmega microcontroller with the GSR framework system. We use a camera module to detect facial expressions in this proposed work. For computing skin conductance, the GSR system with a sensor circuit and the microprocessor is proposed. Smart interfaces and the unique characteristics of human behaviors are required to use these sensors in the field of human-computer interaction.

From Table 2, we have selected a “Grove sensor Galvanic skin sensor. The GSR is one of the most widely used devices for emotion detection. Emotional responses are represented by the GSR output. Sweating gland activity changes in response to changes in our emotional state's intensity. The proposed system's main components are software programs with C-based algorithms, a self-developed ATMEGA328P PCB Board, and a credit card-size portable easily available processor called the Raspberry Pi.

Many fields of human emotional reactions use emotion recognition, such as advertising, sociotechnical appliances, and human–machine interaction. The widespread use of human–computer interaction presents a number of challenges in terms of creating smarter interfaces that match the uniqueness of human behaviors. While choosing GSR we have considered that real-life situations like joyful, scary, dangerous, or emotionally meaningful events stimulate humans sweat gland activity. Sweating exhibits emotional arousal effects, hence with the following combination of facial emotion recognition and GSR can provide better framework as compared to the previous work.

The system works with the following steps:

*Step 1*: Setting up the Raspberry pi

*Step 2*: Using VNC Viewer software for connection establishment

*Step 3*: Coding the program for GSR and Camera for emotion recognition

*Step 4*: Combining setup for GSR sensor and facial expression

*Step 5*: Interpreting values of the GSR sensor for arousal and plotting graphs (This step is explained in the Result section)

*Step 6*: Facial expression emotion recognition on the raspberry pi (This step is explained in the Results section)

The stepwise implementation of the proposed methodology can be described as follows:

*Step 1*: Initialization of the Raspberry Pi

The proposed system employs the Raspberry Pi as a processing unit. In order to activate Raspberry Pi (R pi), first we need to download its operating system (OS) to a PC/laptop from the Raspberry Pi official website. The operating system is transferred from a PC/laptop to the SD card.

Now, we need to connect the SD card to the Raspberry pi via an external cable. The raspberry pi setup is connected to peripherals such as the keypad and mouse

*Step 2*: VNC Viewer and Entering Raspberry Pi Credentials

Now we need network connection to the setup hence, we connect the Raspberry Pi to a network via Ethernet or Wi-Fi. As we are using Laptops, we call the connection as headless or Wi-Fi with remote connections. We use software such as VNC viewer and Advanced IP scanner is used. We used VNC viewer software to connect to the Raspberry Pi over Wi-Fi. The IP address of R. Pi is discovered using an advanced IP scanner. Now we can observe that the VNC viewer displays the screen of the monitor.

**Fig. 1:**
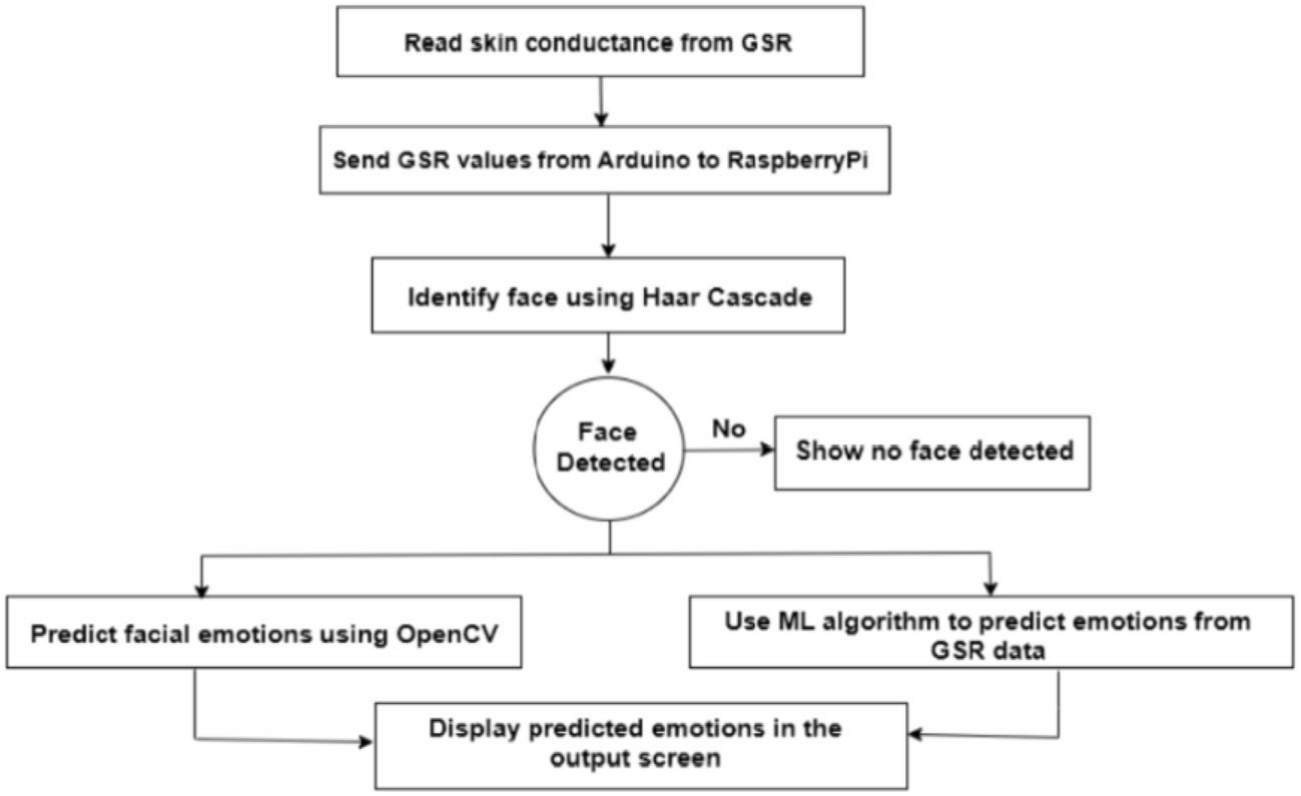
Stepwise process for the implementation of the proposed emotion detection system

Once the Advanced IP scanner scans the available internet-enabled devices around and we can note their ID (Fig. 1).

**Fig. 2:**
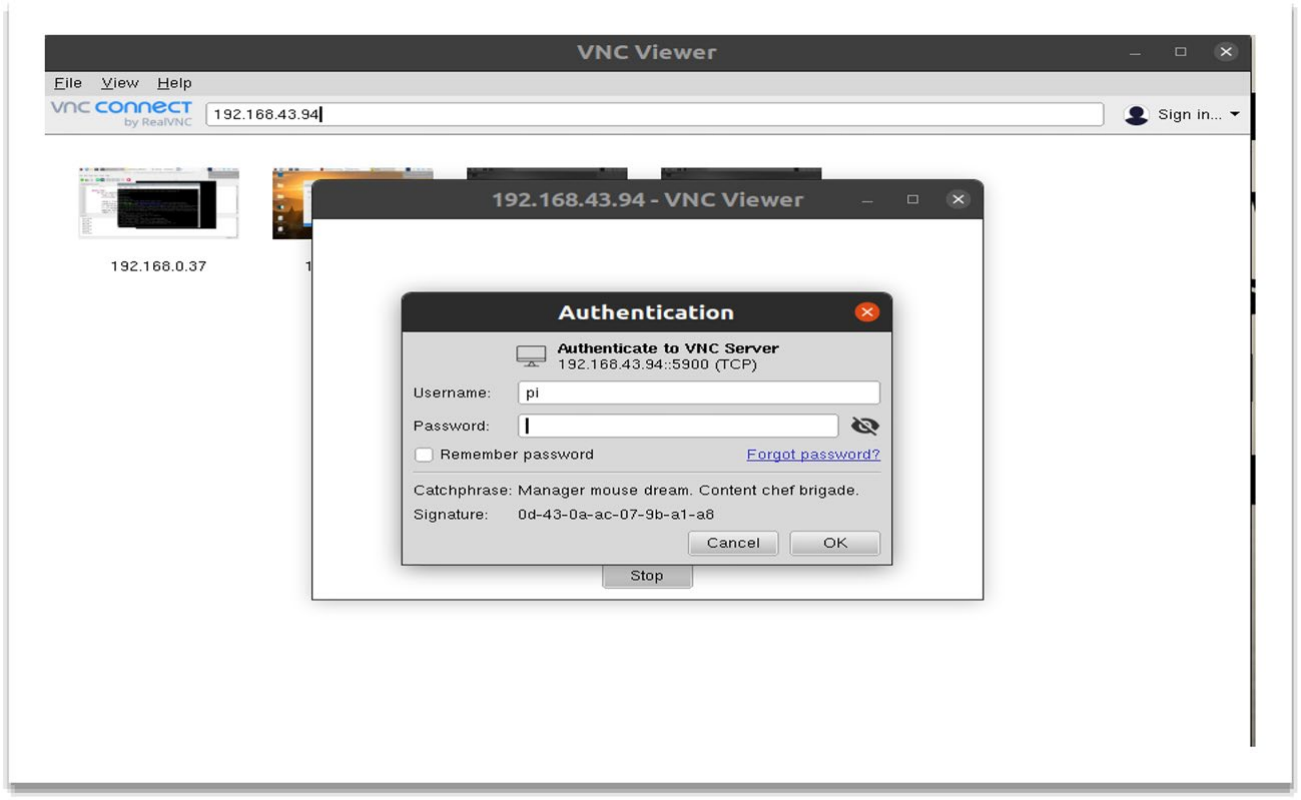
VNC viewer raspberry pi credentials

- We can observe R pi associated with the same Wi-Fi to which laptop is connected.
- We are Noting down Raspberry pi IP Address that just now we observed
- Now we need to Type the same address in the VNC viewer software and connect the Raspberry Pi to the Laptops monitor.
- Using the credentials of the raspberry pi, remotely the network is set up.
- Raspberry Pi Desktop Screen is visible. Figure 2 shows the screen for the credentials of the raspberry pi to VNC viewer

*Step 3*: Coding or loading the programs

**Fig. 3:**
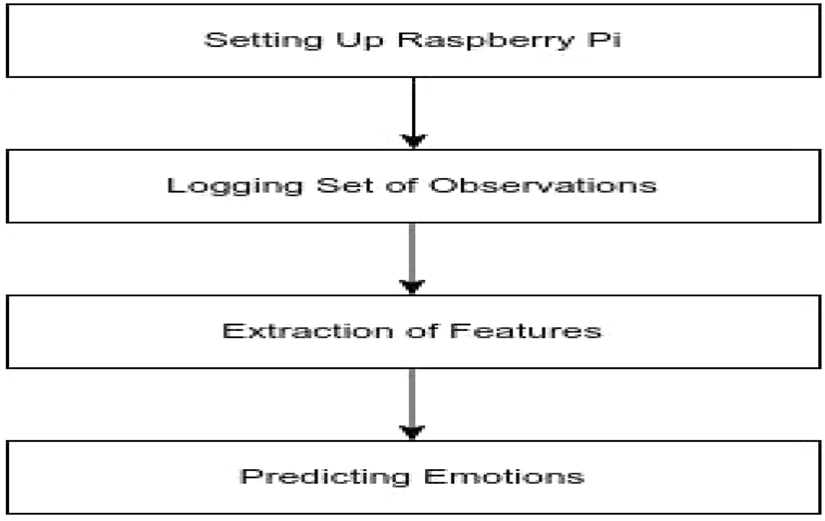
Flowchart for feature extraction and emotion classification

After successfully logging in to the Raspberry Pi, we must code the logic in the Python program that will log the set of observations, display the extracted features, or predict the emotions based on the logic (Fig. 3).

*Step 3.1*: The logic for coding the facial expression part on Raspberry pi

**Fig. 4:**
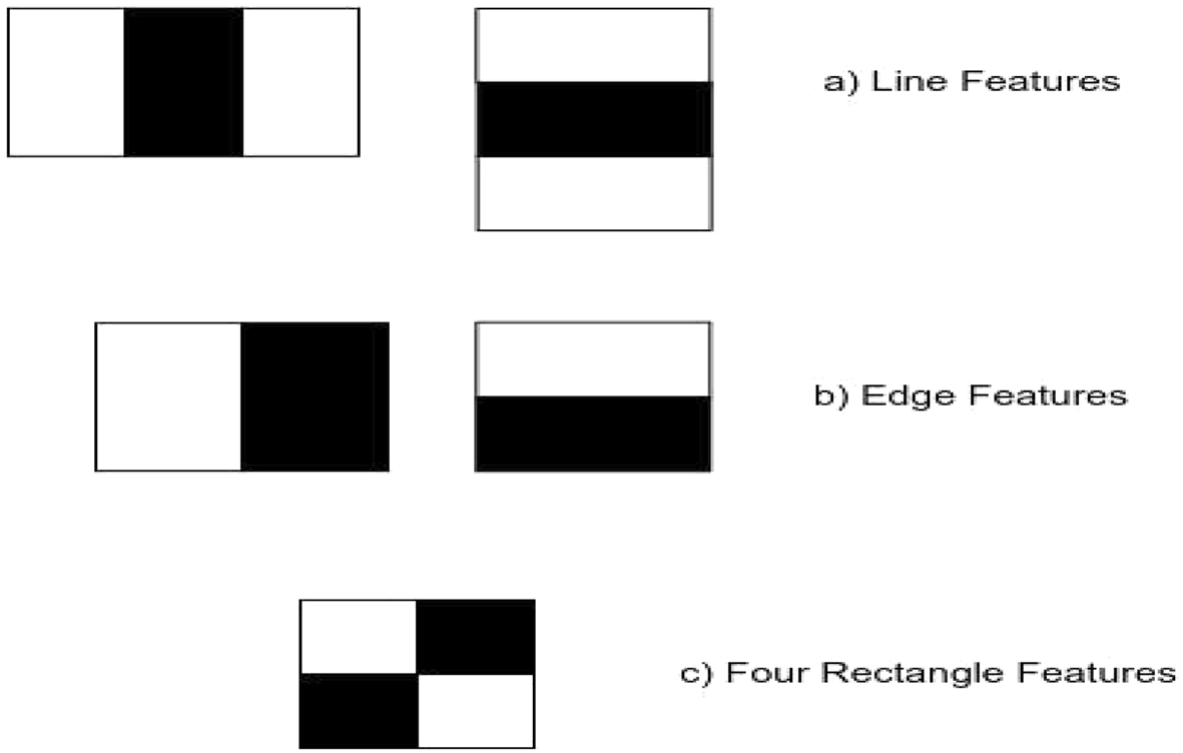
Haar cascade features

The extraction of facial features and prediction of emotions is a critical step. We have used Haar Cascade for facial recognition and OpenCV to extract facial features. Haar Cascade is a face detection algorithm that can detect faces in images and videos. Emotions can be predicted with the help of these libraries. Haar Cascade is a machine learning-based method for training classifiers with a large number of positive and negative images. The Haar Cascade algorithm is divided into four stages (Fig. 4).

*Step 3.1.a* Haar Features Selection

**Fig. 5:**
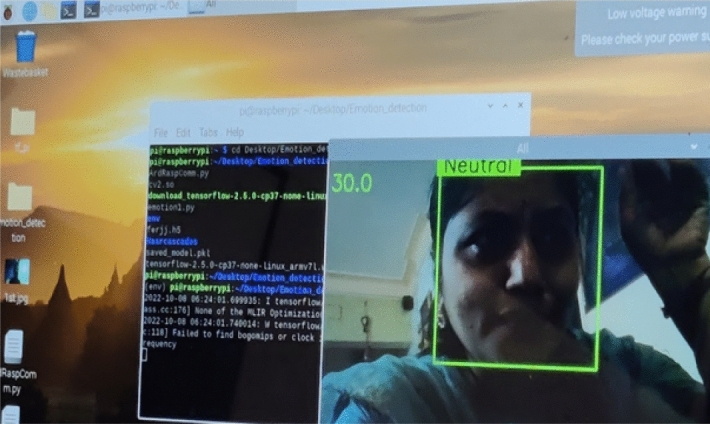
First, the bounding box appears for face detection then neutral emotion recognition appears on the Raspberry pi environment

The nose, eyes, brows, and other facial features must be considered. Haar features, which include Edge, Line, and Four Rectangle features, are relevant for face detection. The horizontal black lines represent the eyebrows (Fig. 5).

*Step 3.1.b*. Creating Integral Images:

This is the intermediate step in which the image is divided into a rectangles array of references.

*Step 3.1.c* AdaBoost training:

There are some decision terms in this stage that split the appropriately and incorrectly classified parts of the image. Using this technique, strong classifiers are formed iteratively.

*Step 3.1.d* Cascade Classifier:

The three previous stages are cascaded, and the Haar cascade is pre-trained with positive and negative images. Positive images are the ones we want to see as our output.

*Step 4*: Coding setup for GSR sensor and Combining facial expression logic to the framework.

**Fig. 6:**
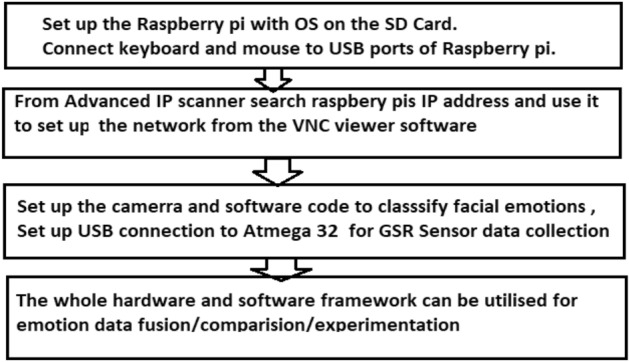
Flowchart for the implementation of the proposed emotion detection system on our prosed emotion recognition framework with portable hardware

Once facial coding is done, we need to record skin conductance from a GSR sensor using an ATmega 32 (Arduino UNO) connected to a Raspberry Pi. A Raspberry Pi camera module is used to record facial emotions. Serial communication is used to send readings from the GSR sensor to the display on the Raspberry Pi. All the combined process till now can be summarized in Fig. 6.

The flowchart for the logic used by our proposed system is shown in Fig. 6. To integrate the emotion recognition systems, the GSR Sensor, Camera Module, and Raspberry Pi are connected. A flex cable connects the camera module to the Raspberry Pi's Camera slot. The Arduino and Raspberry Pi are linked in such a way that they communicate serially and transfer GSR Sensor readings from Arduino to Raspberry Pi in order to process, apply machine learning algorithms, and identify emotions.

**Fig. 7:**
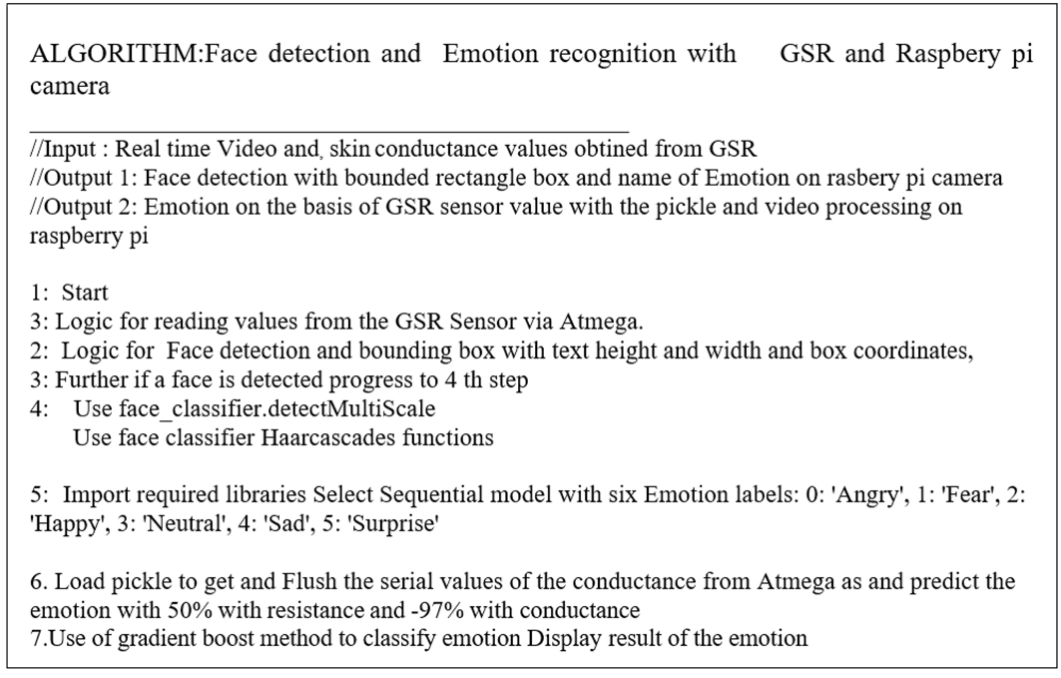
Algorithmic steps for proposed emotion detection with GSR and visual FER method

The algorithmic steps can be summarized as in Fig. 7 as follows.

**Fig. 8:**
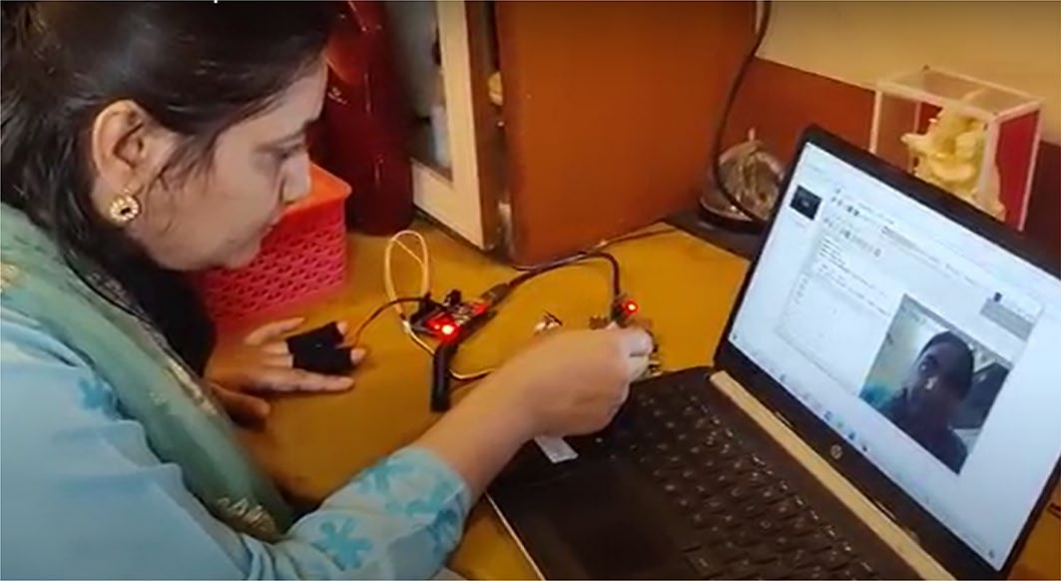
Proposed emotion detection Setup with GSR and visual FER method combined

The data flow starts with the ATmega 32 reading values from the GSR Sensor and sending them to the Raspberry Pi. Then, using a camera module, the facial emotions are recognized, and the face is detected and identified using a popular and widely used Haar cascade method. Furthermore, if a face is detected, OpenCV is used to detect emotions while the Gradient Boosting Classifier algorithm is used to predict emotions from GSR values. Finally, the framework identifies the participant's emotions in a combined manner. The proposed emotion detection Setup with GSR and visual FER method combined can be seen as in Fig. 8.

### Gradient boosting classifier

Gradient Boosting Classifiers are algorithms that create powerful projecting models by combining many weak learning models. They are primarily gradient boosting algorithms. Typically, decision trees are used to increase the slope. Gradient boosting is useful for classifying large amounts of data. The gradient boost classifier combines the AdaBoost method with weighted minimization, after which the classifier is trained. To reduce the loss or difference between the actual and predicted class values, the weighted inputs are recalculated. The process of interpreting gradient boosting involves testing and optimizing the model's parameters until the classifier is accurate enough to satisfy the participant.

## Results and discussion

In this section, we will present the results of our experiments with our proposed framework with following subsections.

**Table:** 

Section	Experimentation	Experiment details
5.1	Experiment 1	Video induction stimuli method
5.2	Experiment 2	Habituation response method with word association/remembering test

**Fig. 9:**
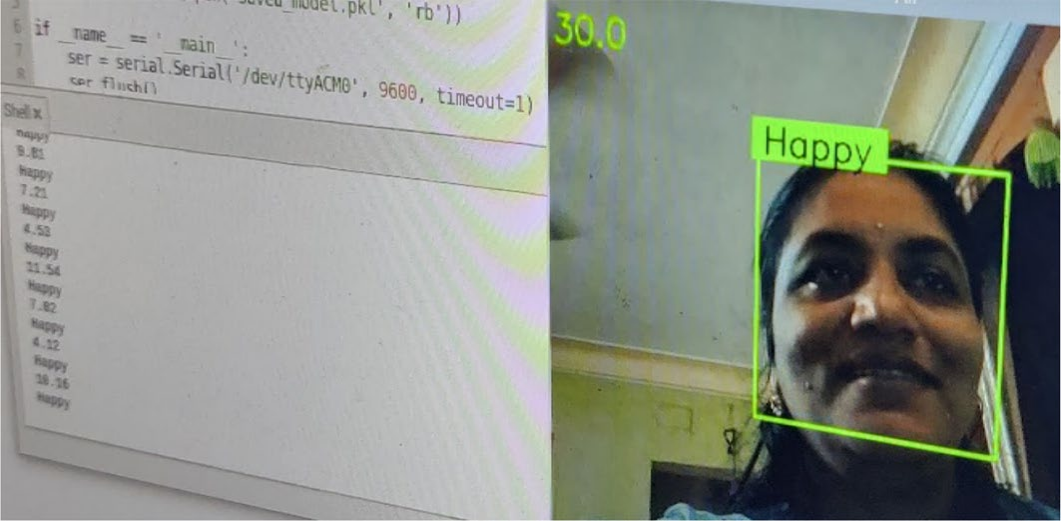
The bounding box for face detection and happy emotion detection on raspberry pi camera and GSR showing skin conductance and happy emotion label and left bottom of the screen

**Fig. 10:**
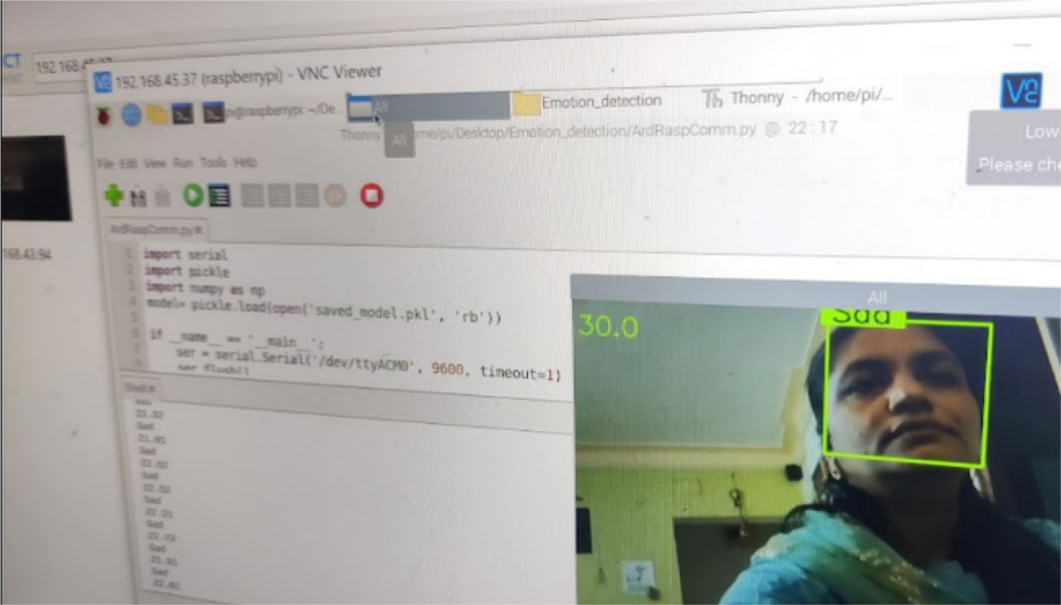
The bounding box for face detection and sad emotion detection on raspberry pi camera and GSR showing skin conductance and sad emotion label and the left bottom of the screen

**Fig. 11:**
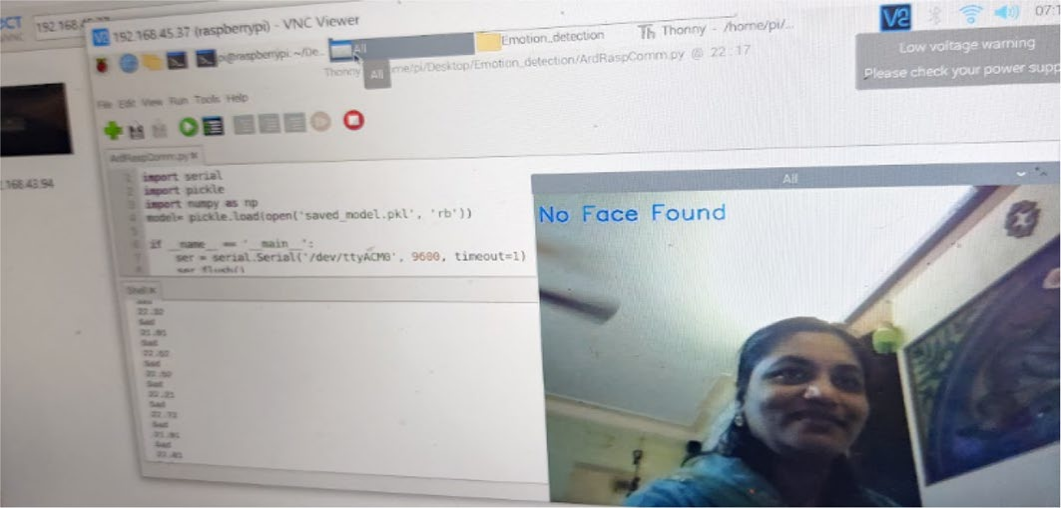
The bounding box does not appear for face detection so no face detected and no emotion detection on the raspberry pi but the GSR method is working and showing skin conductance with the corresponding label

In previous section, we have observed the results of emotion recognition (Refer Figs. 8, 9, 10 and 11) with our raspberry pi and GSR-based framework. It is clear from these figures we can observe both facial emotion recognition and GSR results in a single window.

### Experiment 1: Video induction stimuli method

**Fig. 12:**
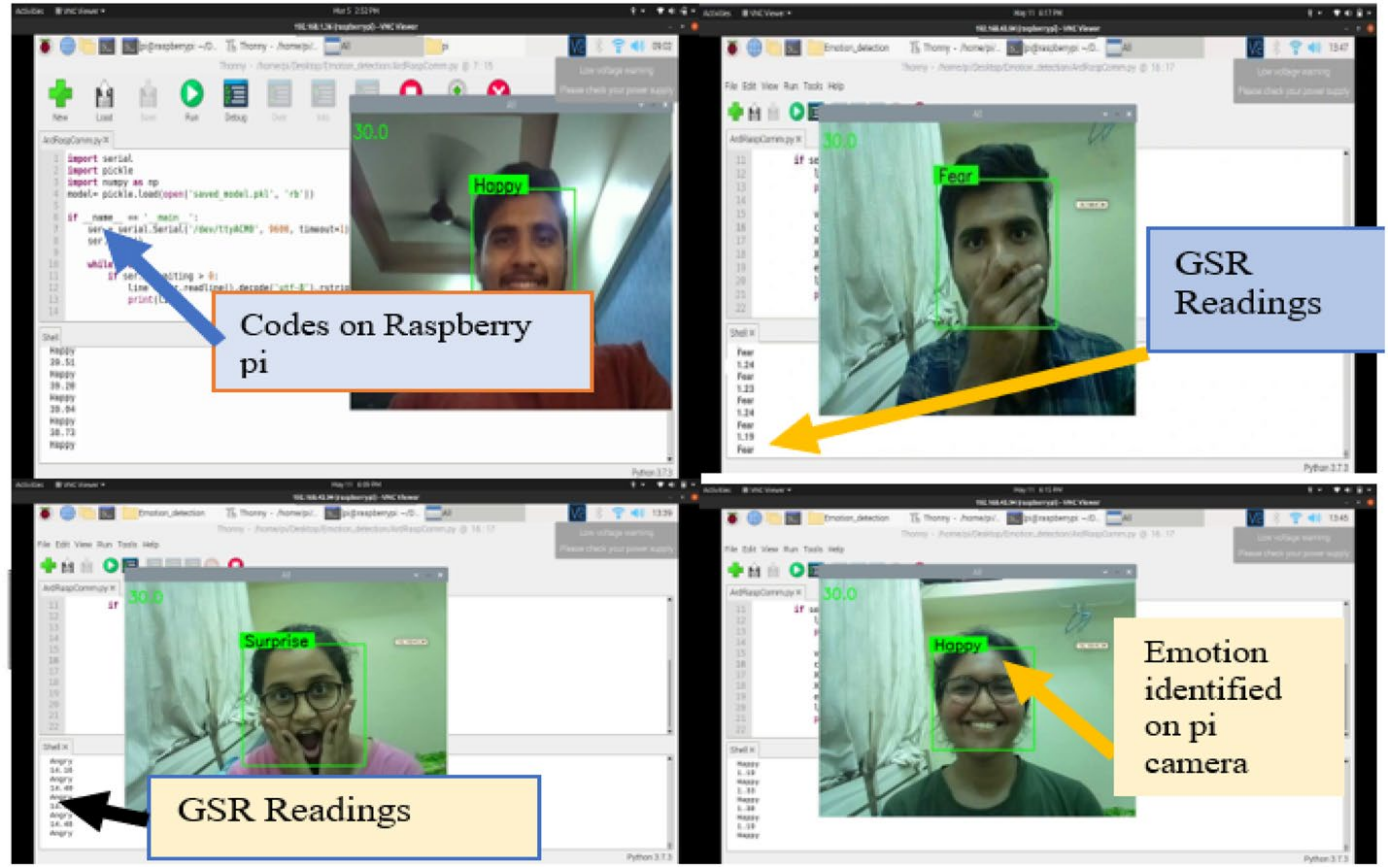
The overall appearance of the screen when the participant is experiencing diverse video stimuli in laboratory conditions (4 different screens captured shown all in one)

Figure 12 depicts the overall appearance of the resultant screen when the participant is exposed to a variety of video stimuli in the laboratory condition.

**Fig. 13:**
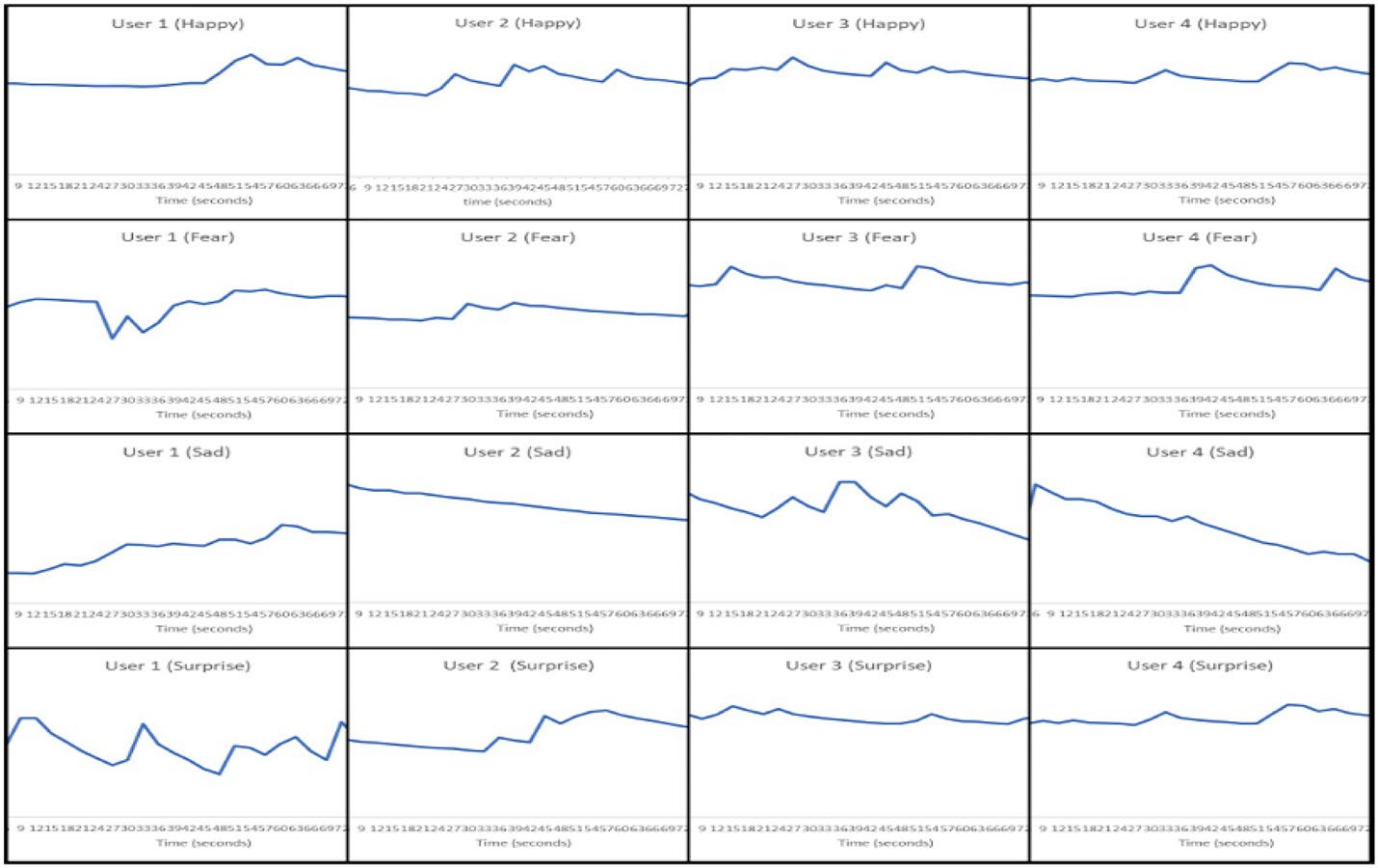
Experimentation to capture the patterns of GSR output on different participants with stimuli defined in Table 1

Stepwise Video induction method can be summarized as follows:

- Participants are requested to be in a relaxed state and to sit in a particular environment. After 10 min in the relaxed condition, the participants will be asked to sit in front of our raspberry pi and GSR-based framework setup (as shown in Fig. 8).
- The only concern for the experiment is the time needed to prepare the setup. Each participant needs five to ten minutes to wash their hands and apply the gel to their index and ring/middle fingers. Ten to fifteen minutes time is needed for each participant to reach a relaxed starting state. We have chosen 4 participants for experimentation which are authors of the article. The experimentation is done adhering to Covid 19 guidelines and regulations.
- In a single day, only one emotion video is shown to each participant. For example, the first as a stimulus is happy video. The clip is about marriage ceremony of Cricket star Virat Kohli and Bollywood star Anushka Sharma. This clip is shown the results of the experimentation of each type of emotion is recorded in terms galvanic skin sensor emotion matrix 1 (Fig. 13) and matrix 2 of facial emotion matrix (Fig. 22)
- For each emotion in Table 3, step 1 to 3 are repeated. The careful arrangement of readings in terms of GSR readings and FER is arranged in the form of emotion matrices.

- Matrix 2: Galvanic skin sensor emotion matrix (Fig. 13)
- Matrix 2 of facial emotion matrix (Fig. 22)

e. The results of the observation are displayed in columns titled Results (Table 2). In addition, the outcomes of emotions acquired by the Raspberry Pi camera are expressed as an emotion matrix as shown in Fig. 13.
f. The galvanic skin Response and corresponding graphs and inferences from the graphs s are shown in Figs. 15, 16, 17, 18, 19, 20, 21 and 22.

**Fig. 14:**
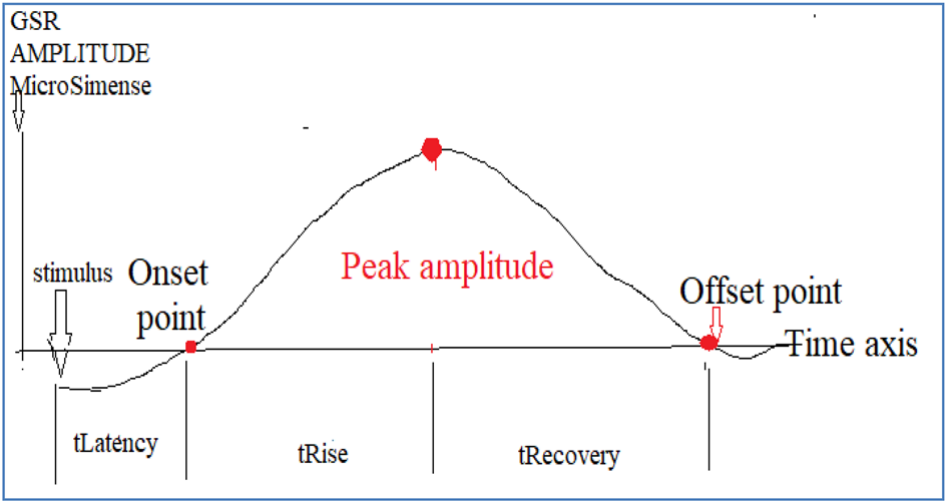
General pattern of GSR output [17]

**Table 3:** Experimentation for capturing arousal GSR sensor and facial expressions with the raspberry pi camera

Experimental arrangements				Results		
S. no.	Video No and Link	Gender of the participant whose emotions captured	Place where emotions captured	Emotion observed (Via GSR)	Emotion observed (Via Facial Expressions)	Participant 1 skin conductance in micro siemens during watching the video at random moment
1	[1] https://www.youtube.com/watch?v=JNKZN8uq1H8 : Virat and Anushka wedding video	Participant 1 in column 1 is woman, participant 3and 4 in column 3 and 4 are girls and the participant 4 in column 2 is boy	All responses are taken in controlled conditions where subject is allowed to relax 10 to 15 min and then after 5 s the videos are observed by the participants and observation are captured via our setup	Arousal as per subject response compared to baseline is shown in the following graphs 13 to 20	*Happy expressions* As shown in the emotion matrix of Fig. 9 row1	26.42
2	[2] https://www.youtube.com/watch?v=cVSICYyPbGY : Sad Emotional story				*Sad expressions* As shown in the emotion matrix of Fig. 9 row3	35.96
.3	[3] https://www.youtube.com/watch?v=vSidTZlSbzY : World's Scariest Pranks…				*Fear expressions* As shown in the emotion matrix, of Fig. 9, row 2	29.63
4	[4] https://www.youtube.com/watch?v=0gx6sNYUJRQ : Pharrell Williams on TV show "Surprise Surprise"				*Surprise expressions* As shown in the emotion matrix of Fig. 9, row4	22.89

**Fig. 15:**
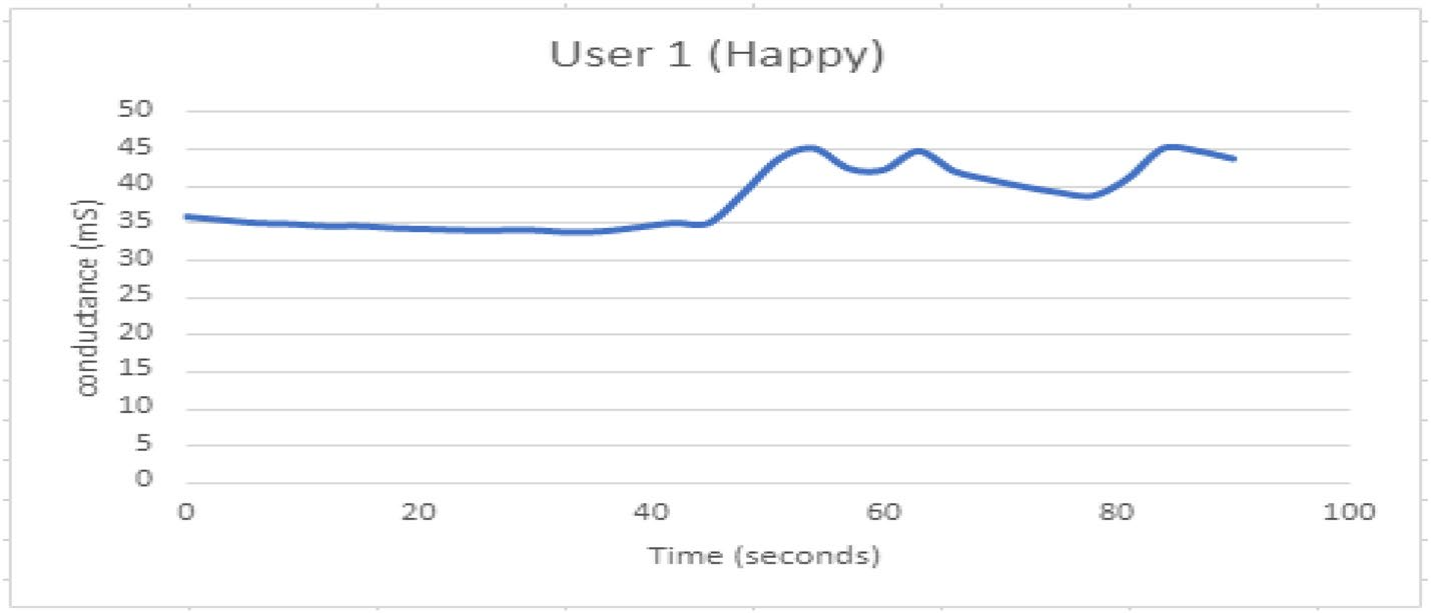
GSR response when participant 1 is Happy

Table 3 lists the video induction stimuli and their effects. The effects of stimuli are discussed in sections "Galvanic skin responses" to "Correlation matrix", which are listed below.

#### Galvanic skin responses

Figure 12 depicts the combined effect of GSR and facial expression responses onscreen. Figures 13, 14, 15, 16, 17, 18, 19, 20, 21 depict galvanic skin response graphs.

#### Facial expression responses

Effects of GSR and facial expression responses on the screen are shown in Fig. 12. Figure 22 depicts the representation of four participants' facial expressions in terms of the Emotion matrix on the framework of the Raspberry Pi camera.

#### The output of the GSR in terms of the skin conductance and resistance

 Tables 3 and 4 show the output values of skin conductance.

**Table 4:** Readings of the GSR Method for video induction method

S. no.	GSR avg	Resistance in ohms	Conductance mho	Video	Gender
1	158	37,853.10	26.42	1	F
2	196	40,514.28	35.96	4	M
3	131	33,753.28	29.63	3	F
4	98	31,652.39	22.89	2	F

The statistics of the observations for the video induction are summarized in Table 5

**5.1.4 Correlation matrix: **In this section, the values of the correlation matrix are shown.

The detailed explanation of each of the above points is as follows:

#### Galvanic skin responses

The GSR is known as the electro-dermal response because skin conductance responds proportionally to physiological internal or external stimuli. This experimentation with different stimuli is resulting in different levels of arousal. Arousal and valance are not indicators of emotion recognition, but rather of memory and attention to specific phenomena in response to stimuli. When someone is startled by stimuli such as bright light or loud noise, the electro-dermal responses can last up to 3 s. The first response to a sudden start is quite strong, whereas later stimuli do not produce large responses due to the habituation effect. The sudden startle, however, can be reactivated after the subject has relaxed. Experiment with stimuli defined in Table 1 to capture patterns of GSR output on different participants.

The above experimentation and GSR responses can be grasped with an understanding of the following theoretical background. Internal body parameters such as heartbeats, pulse rate, and sweating change as skin conductance increases. Skin conductance is measured in microsiemens or micromhos. Arousal is detected as sweat glands increase the skin conductance response. The experimentation done by us with different video induction stimuli is resulting in different levels of arousal. Arousal and valance are not indicators of emotion recognition, but rather of memory and attention to specific phenomena in response to stimuli.

The important aspect of GSR arousal detection is that it cannot distinguish between positive and negative arousal; thus, to confirm the exact emotion at the time of the arousal, we used facial expression recognition with the raspberry camera. The arousal level visualization can be seen from the galvanic skin response graphs in Fig. 13).

The general pattern of galvanic skin response is shown in Fig. 11. The response of changes in skin conductance is seen after the stimulus is provided to the subject/participant (the person whose response is to be recorded). The response is having on x-axis time and on y-axis amplitude in microsiemens. The response is divided into 3 parts as follows:

- *Latency time period*: The time lag from which stimuli is provided to the subject/participant to the time period when there is a change in the skin conductance response is observed is called the latency time period.
- *Rise time*: The time required from the onsite point to the peak amplitude point is called the rise time. This time period is also called as a phasic response. Sometimes this is referred as skin conductance response. Because the tonic level can be influenced by factors other than emotion, we must focus on the phasic response.

Some subjects/uses have not responded well to the baseline and are referred to as stables. Subjects/participants who exhibit peaks with high amplitude arousal are referred to as labile. Although the phasic response does not make a positive or negative statement, it does provide arousal intensity. However, in order to obtain an exact correspondence, we combined GSR output with visual FER.

Figure 15 depicts the GSR response when participant 1 is happy. The response indicates a longer recovery time, with the onsite point observed at 40 s.

**Fig. 16:**
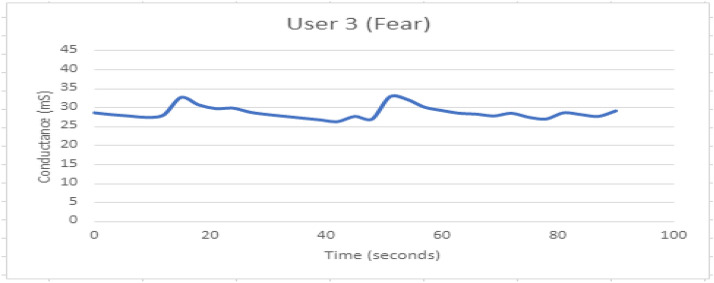
GSR response when participant 3 is experiencing fear

Figure 16 shows GSR response when participant 3 is experiencing fear. The rise time appears less.

**Fig. 17:**
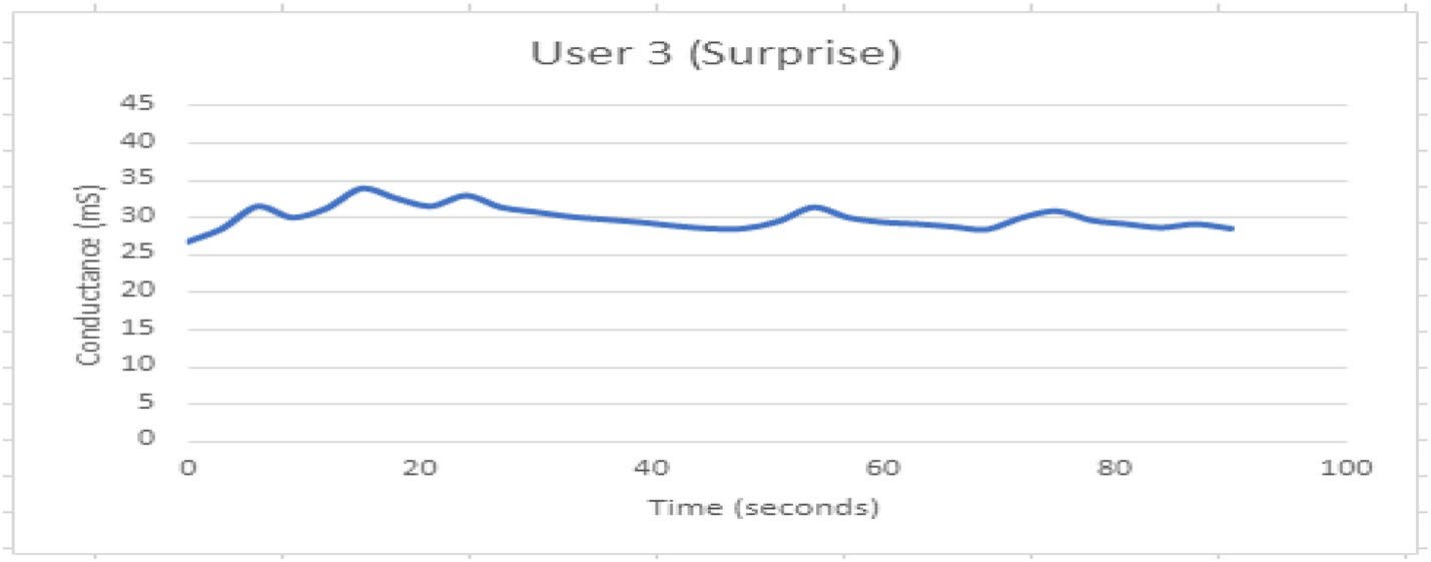
GSR response when participant 3 is experiencing Surprise

The pattern is non-repetitive but a habitual reflex is observed. The first arousal at 18 s is having highest peak as compared to the next peaks.

**Fig. 18:**
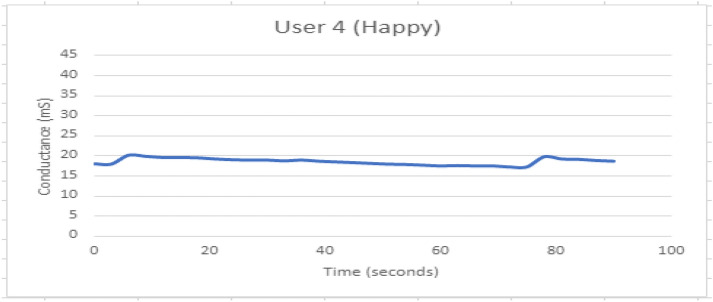
GSR response when participant 4 is experiencing Happiness feeling

Figure 18 shows GSR response when participant 4 is experiencing Happiness feeling. The response appears to be stable.

**Fig. 19:**
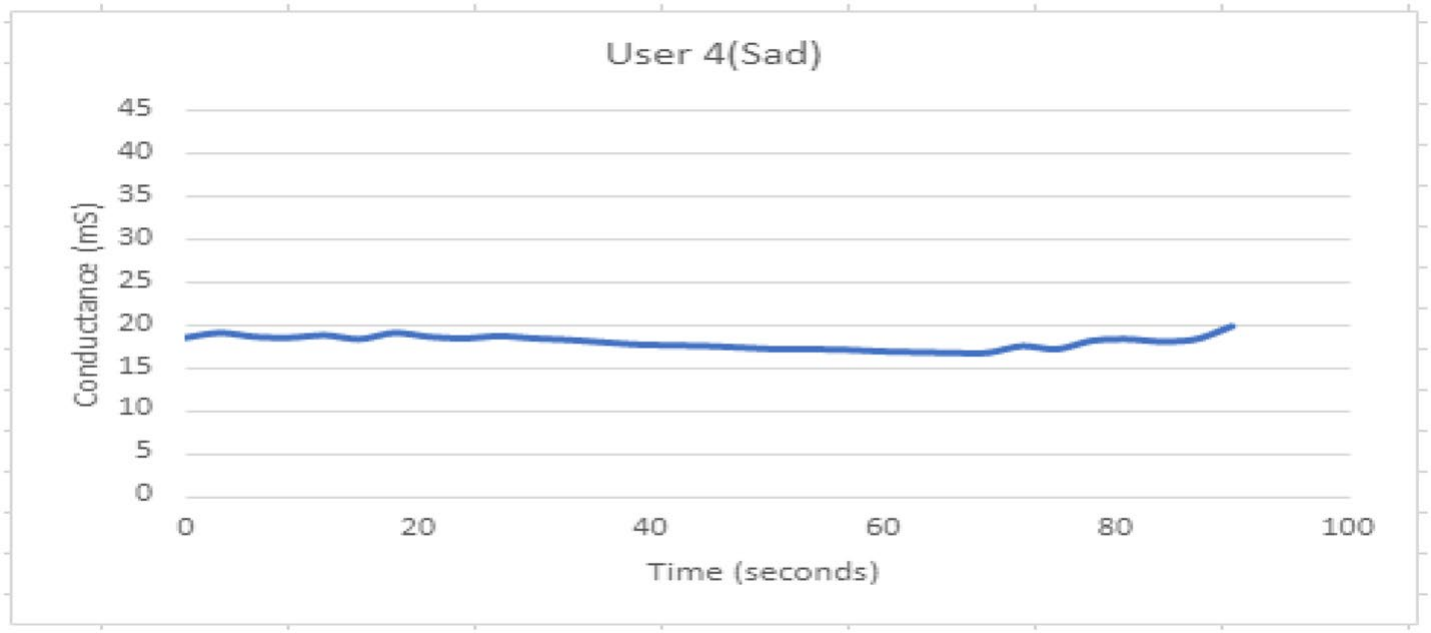
GSR response when participant 4 is experiencing sadness

Figure 19 shows GSR response when participant 4 is experiencing the sad feeling. The response appears to be stable.

**Fig. 20:**
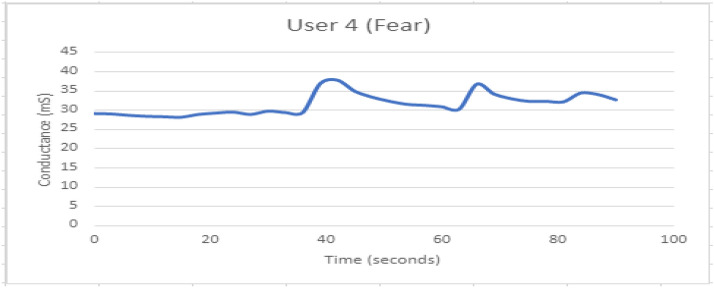
GSR response when participant 4 is experiencing fear. The response looks to be stable

The values of the GSR sensor parameters are also recorded while the graphs are plotted. Table 2 includes the participant's gender, the GSR average (avg.), the participant's resistance and conductance, the category number for the video selected from Table 2, and the participant. The average value of ten GSR readings is known as the GSR average.

#### Facial expression responses

The real-time results of emotion recognition on raspberry pi hardware for four participants (authors of this paper) are expressed as an emotion matrix and a GSR output matrix under controlled conditions, as shown in Table 3.

#### The output of the GSR in terms of the skin conductance and resistance

Readings of our proposed setup are listed in Table 4. To code the program in Embedded C we have used, the Resistance (*R*) and conductance $(\rho)$ formulae from the datasheet in Eqs. 1 and 2. The statistics of the observations are summarized in the following Table 5.

**Table5:** Statistics of the GSR observations of the subjects for skin conductance and resistance

Parameter	GSR avg	Resistivity Ω (× 10^3^)	Conductivity (*⍴*)
Count	1359.0	1359	1359.0
Mean	172.44	1.9	26.85
Standard value	113.53	9.3	12.55
Min	0.00	2.0	0.50
Max	521.00	2.0	50.00

1 $${\text{R}}~ = \left( {1024 + 2 \times Gsr_{{{\text{average}}}} \times 10,000} \right) \div {\text{Abs}}\left( {512 - {\text{gsr}}\_{\text{average}}} \right)$$

2 $$\rho=1/\mathrm{Resistance}*1000000$$

Table 5 displays the GSR reading statistics. The ‘count ‘row displays the total number of sample observations collected. The mean row shows the average of each column, which is GSR average, resistance (Ω), and conductance (*⍴*). The skin conductance, resistance of the participants and std. value row (of Table 5) represent the dataset's important values. The min and max rows represent the minimum and maximum values of particular input parameters.

#### Correlation matrix

The correlation matrix represents the correlation of the parameters on the scale of 0 to 1. The correlation matrix is observed as shown in Fig. 23. For video induction experimentation, GSR avg., resistance, conductance, video category, and gender type are taken into account.

- There is a negative correlation between conductance and resistance.
- The correlation between conductance and gender has a value of 0.5.
- Gsr avg. and resistance have a correlation of 0.5.

**Fig. 21:**
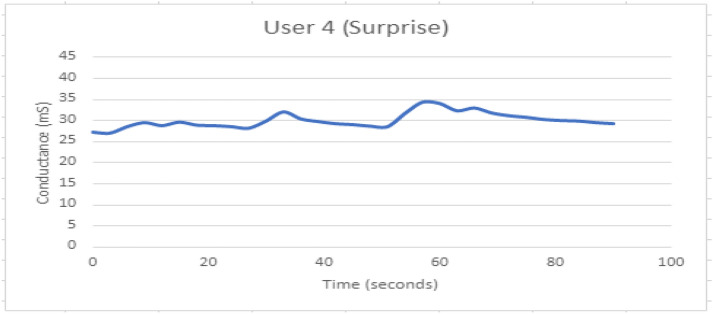
GSR response when participant 4 is experiencing Surprise

**Fig. 22:**
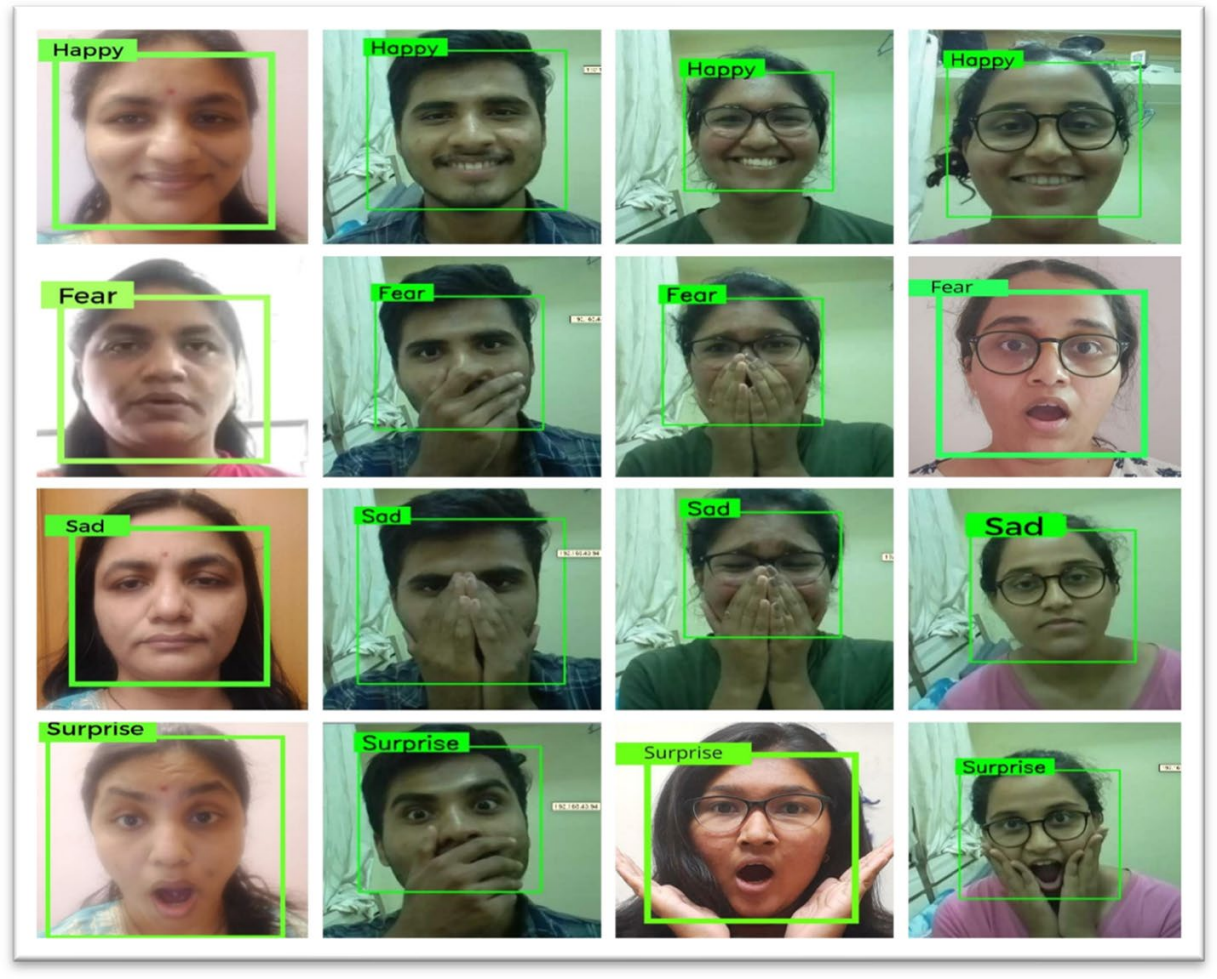
The representation of facial expressions in terms of the emotion matrix of four participants identified on the framework of the raspberry pi camera

**Fig. 23:**
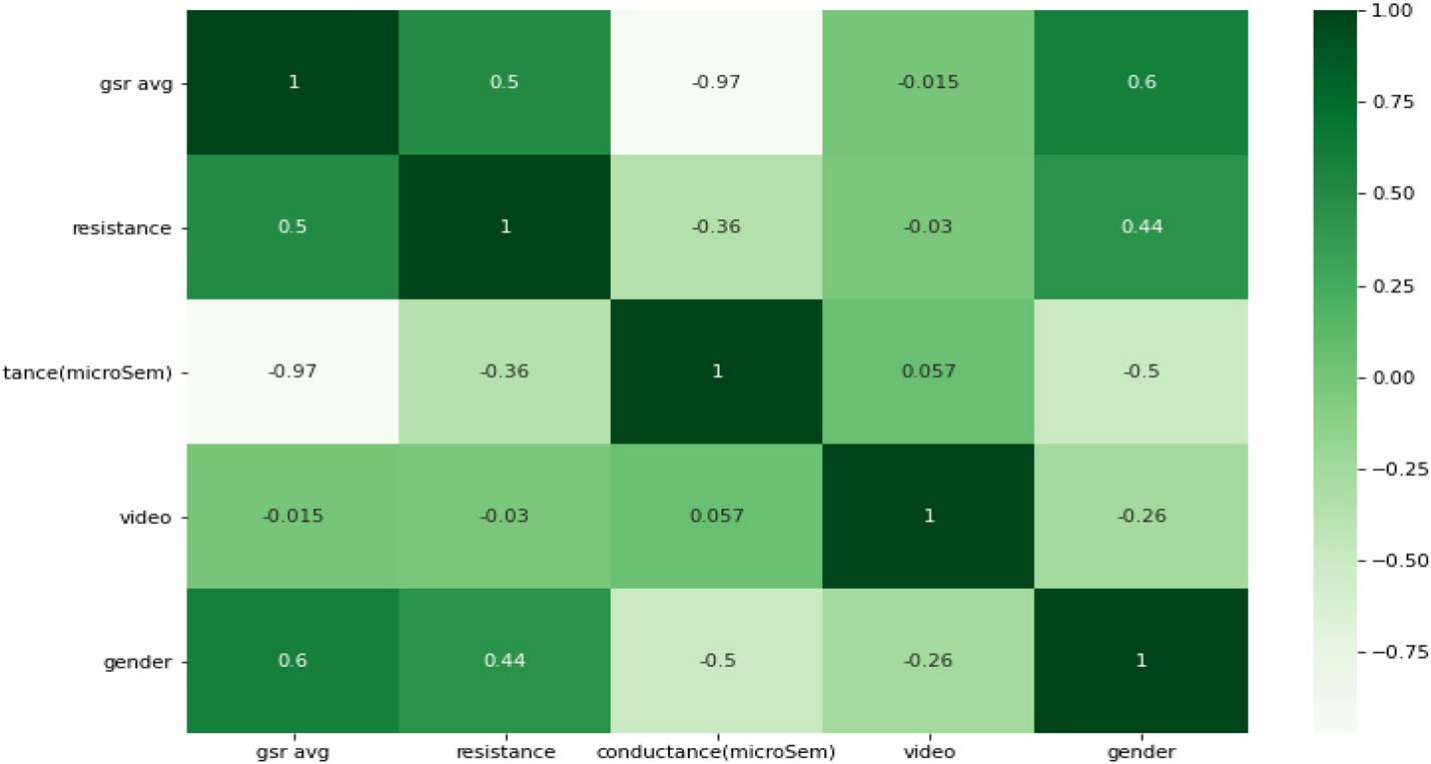
Correlation matrix for GSR avg., resistance, conductance, video, and gender values from the correlation matrix. Following are the observations

### Experiment 2: Habituation response method with word association/remembering test

*Habituation response method with word association/remembering test*

Before putting the description of actual experimentation of habituation, let us be familiar with the concept of the habituation. Habituation means to become familiar with the effects and triggers after certain epochs.

Let us see the habituation effect by some examples. It is observed that when someone is startled by stimuli such as bright light or loud noise, the electro-dermal responses can last up to 3 s. The first response to sudden stimuli is quite strong. Later, after some time, stimuli do not produce large responses due to the habituation effect. The stimuli used for the sudden startle can be reactivated after the subject/participant has relaxed. Experiment with stimuli (defined in Table 3) can be used to capture patterns of GSR output on different participants. This physiological test is known as the habituation test experiment.

**Our setup arrangement for habituation test experiment**

We have used our galvanic skin sensor-based framework for habituation test experiment. Participants are not required to look at the camera. The test requires different time periods for different participants. For habituation test, it is better not to use facial emotion recognition. This is because habituation test requires mental concentration of the participants and observers.

- The participants are instructed to seat quietly and relax.
- The participants are asked either about word associations or asked to remember conversation with Friend and write it down.
- At first time instance, participants are very attentive.
- After certain specified time period, again the same experiment of word associations or remembering conversation with Friend and write it down is repeated.
- The participants start losing interest and focus. Finally, the subject/participant would have no effect on subsequent occurrences of the same stimuli.
- The time when they lose complete interest is subjective and is called as habituation time period. Table 6 shows the complete statistics of the GSR observations of the subjects for skin conductance for the habituation test experiment.

**Table 6:** Statistics of the GSR observations of the subjects for skin conductance and resistance

	Stimuli	Skin conductance An instance of first time occurrence	Skin conductance An instance of second time occurrence	Skin conductance An Instance third time occurrence	Time for habituation no changes (s)
1 (female)	Remembering Friends conversation	45.65	44.24	40.00	100
2 (male)	Remembering Friends conversation	55.01	53. 1	49.11	70
3 (female)	Word association	39	27	18	150
2 (male)	Word association	50.00	45.221	32.33	270
4 (female)	Remembering Friends conversation	44	33	24	130

**Observations of Habituation test**:

- The male participant's word association test results show more time.
- The female candidate is seen to have the largest habituation time to remember friend’s conversation for the other four candidates.
- Ever participant's skin conductance decreases over time, and every subject's skin conductance value is highest during the initial application of stimuli.

Habituation test experiment is useful for behavioral traits, personality test. The test is useful for individuals behavior in certain situations. However, the bottom line is habituation effect is completely subjective and depends on individual’s ability to respond and time to retain the effect of stimuli.

## Conclusion

We have successfully done successful implementation and testing of the combination of real-time human emotion recognition using skin conductance signals and facial expression signals on portable hardware. This framework is implemented and experimented with for video induction and habituation experiments. The framework is designed to run on the portable hardware of Raspberry Pi and Arduino with machine learning techniques for successfully recognizing emotions. The illuminating aspect of the proposed method is the selection factor for Grove galvanic skin sensor from the currently available trademarks. The same framework used for conducting video induction and habituation experimentation.

The research contribution of our work is that our method provides a real-time portable of GSR and computer vision-based framework with video induction and habituation experimentation. The other contributing factor of our method is the step-by-step comprehensive documentation of the suggested framework.

### Research contribution

1. There are several articles that describe emotion recognition using sensors, as well as articles on facial emotion recognition. Most available methods provide dataset simulations, but this work has better prospects in terms that it displays the results of the fusion of computer vision and sensor methods on a single screen. The proposed configuration is a first-of-its-kind real-time Hardware implementation of galvanic skin biosensor sensor and visual signals. For our proposed setup/framework, we propose the term sensovisual approach.
2. A notable contribution of the proposed method is that both real-time camera and GSR sensor values (converted to emotion class label) are visible on the same screen; thus, the outputs of both methods can be visualized simultaneously on the same screen. We have done extensive market survey of GSR modules, we found our way of representation of facial emotion recognition and galvanic skin sensor values with the emotion class label is the unique and is useful for finding any difference in facial and sensor-based reading.
3. When compared to the commercial tradename models listed in Table 2, our proposed module offers greater programming flexibility at a lower cost, as well as a portable low-space module.
4. The GSR sensor in our setup is similar to the polygraph test setup. However, the proposed setup outperforms and distinguishes itself from traditional polygraph tests in the following ways. The person's facial expression is monitored on a remote monitor during a polygraph test without the subject’s/participant‘s knowledge. However, in the proposed setup, the subject/participant can simultaneously observe both the facial and skin sensor readings and automatically capture emotions. The benefit of our proposed setup is that if the subject/participant tries to hide his or her emotions, the combined results of biological and facial expressions are automatically recorded. The participant can manipulate the facial emotions, but not skin conductance values.
5. Despite of several hardware implementation problems, the effective implementation of real-time affective computing is done on the hardware

### Limitations

- Setting up experiments with our approach requires longer time than plug-and-play commercial trademark galvanic skin sensor modules. But our method offers advantage of programming flexibility as well as real-time sensor and vision (facial expressions) on the same screen.
- Limitation of facial emotion recognition is that if the camera is unable to detect boundary box around the face, then the emotions of participants cannot be captured.
- Another limitation of the setup is that if there are multiple faces in front of the camera, then only the person in front of the camera is considered as the subject.
- In some cases, when the operator comes in front of the camera by accident, the camera captures the operator's emotion. At the same time, GSR is linked to the subject’s/participant‘s hands. This situation leads to misinterpretation of the operator's emotions based on visuals and sensor readings from the subject’s/participant‘s body parameters.

### Advantages

This study provides a systematic guide to the implementation of face and emotion recognition on the Raspberry Pi. Aside from documentation, the experimentation is useful for combining sensor methods, and visual methods and having fusion of emotions recognition methods. Emotional arousal can be measured using physiological parameters and GSR sensor implementations. The GSR sensor readings do not indicate positive or negative variations, but they do provide an indication of arousal due to changes in the subject’s/participant‘s skin conductance caused by the stimuli provided in the controlled environment. The facial expression method and the galvanic skin response method are both complementary.

The graphical analysis of the GSR method reveals that some participants are stable and do not respond to some video stimuli with skin conductance. Visual inspection of face detection and facial expression on the raspberry pi is useful for emotion recognition in such cases.

In some cases, antisocial social elements like criminals may be expert in hiding emotions on faces. If a person is not showing facial expressions but there is arousal found in skin conductance, the person may show true expressions after a certain period of time and thus may be closely monitored. This study's combination of physiological and facial expression-based emotion recognition is implemented and tested successfully on a portable Raspberry Pi device.

### Applications

Computer technologies in the field of human-computer interaction are now widely used. Emotion recognition is a popular topic in many fields that use human emotional responses as a symbol for advertising, sociotechnical appliances, or human-machine interactions. Potential applications include lie detection devices and screening at airports and entrances. Identification of potential customers in marketing fields [17], pain management, the launch of new entertainment products and technological appliances such as human-robot interaction, and so on.

### Future scope

Our setup also has the potential to be implemented for the diagnosis of disorders associated with perspiration, such as epilepsy, diabetes, and bipolar disorder. Sweat and temperature can be used as the basis for the design and development of a fact checker. The Raspberry Pi is a tiny computer, but it has the capacity to be networked in a system, which means it could be used for some of the future work related to emotion transmission on Networks. Commercial polygraphs, lie detector, feedback tester, and market surveying machines are possible applications of our setup.

## Data Availability

Due to privacy and ethical concerns of participants (though authors are the participants, they do not want to declare the emotions observed as a subject while experimentation. In paper participant 1, 2, 3, 4 is written), neither the data nor the source of the data can be made available.
